# New Insights on Human Polyomavirus JC and Pathogenesis of Progressive Multifocal Leukoencephalopathy

**DOI:** 10.1155/2013/839719

**Published:** 2013-04-17

**Authors:** Anna Bellizzi, Elena Anzivino, Donatella Maria Rodio, Anna Teresa Palamara, Lucia Nencioni, Valeria Pietropaolo

**Affiliations:** ^1^Department of Public Health and Infectious Diseases, “Sapienza” University of Rome, P.le Aldo Moro, 5-00185 Rome, Italy; ^2^Department of Public Health and Infectious Diseases, Institute Pasteur, Cenci-Bolognetti Foundation, “Sapienza” University of Rome, P.le Aldo Moro, 5-00185 Rome, Italy; ^3^San Raffaele Pisana Scientific Institute for Research, Hospitalization and Health Care, Via Val Cannuta, 247-00166 Rome, Italy; ^4^Sbarro Institute for Cancer Research and Molecular Medicine, Center for Biotechnology, College of Science and Technology, Temple University, 1900 N. 12th Street, Philadelphia, PA 19122, USA

## Abstract

John Cunningham virus (JCV) is a member of the *Polyomaviridae* family. It was first isolated from the brain of a patient with Hodgkin disease in 1971, and since then the etiological agent of the progressive multifocal leukoencephalopathy (PML) was considered. Until the human immunodeficiency virus (HIV) pandemic, PML was rare: in fact HIV-induced immunodeficiency is the most common predisposing factor accounting for 85% of all instances of PML. This data led to intense research on JCV infection and resulted in better understanding of epidemiology and clinic-pathologic spectrum. Recently, cases of PML have been observed after the introduction of monoclonal antibodies, such as natalizumab, rituximab, efalizumab, and infliximab, in the treatment of autoimmune disease, underlining the important role of host immunity in PML pathogenesis. In this review current understanding of the JCV infection and the new findings relating to the pathogenesis of PML has been comprehensively revised, focusing our attention on the interaction between the cellular and viral molecular pathways implicated in the JCV infection and the modulating role of host immune surveillance in the viral reactivation from a latent state.

## 1. Introduction

John Cunningham virus (JCV) is a circular double-stranded DNA polyomavirus isolated in 1971 from the brain of a patient with Hodgkin disease [1], and it is the etiological agent of the progressive multifocal leukoencephalopathy (PML), first described by Åström and colleagues in 1958 [2]. JC is an ubiquitous, neurotropic virus: in fact, blood samples taken from healthy individuals indicate that 50–90% of adults have been exposed to this virus, with 19–27% of those people shedding JCV in their urine. The seroprevalence increases with age but acquisition of this virus is not associated with a clinical syndrome [3, 4].

A N-linked glycoprotein with an *α*-(2,6)-linked sialic acid is one of the cellular receptors for the virus. Additionally, JCV can bind to the serotoninergic 5-HT_2*α*_ receptor to infect astroglial cells *in vitro* and kidney epithelial cells, B lymphocytes, and glial cells *in vivo.* Nevertheless, it is difficult to propagate the virus in cell cultures [3].

Viral DNA is transcripted on both strands and it encodes for the early genes counterclockwise and for the late genes clockwise. The early proteins are involved in viral transformation, gene regulation, and replication, whereas the late proteins are the viral capsid proteins. The agnoprotein is a late protein associated with DNA damage and interferes with DNA repair mechanisms [5]. The coding region is well conserved and is associated with the various subtypes that can be found in different geographical areas. Conversely, the noncoding control region (NCCR) sequence is hypervariable and contains determinants for neurotropism and neurovirulence. The rearranged NCCRs, formed during immunosuppression, correlate with poor clinical outcome in patients with PML [4, 6].

PML is a demyelinating disease of the brain that affects adults and rarely children. Patients present neuropsychological deficits at PML onset. The natural disease course is progressive and leads to death within months if the patients remain immunocompromised. PML was originally recognized as a rare complication of hematological malignancies or systemic inflammatory disorders: however, a dramatic 50-fold increase in the incidence occurred with the HIV epidemic. Moreover, PML can be seen after organ- and stem cell transplantations and, recently, in patients under treatment with immunomodulatory compounds like monoclonal antibodies. As a result, physicians from many disciplines are now likely to encounter what was once a rare disorder [3, 7]. Finally, CD4^+^ and CD8^+^ T lymphopenia seems to be the primary PML risk factor, following the use of natalizumab (Tysabri; Biogen Idec, Elan Pharmaceuticals), efalizumab (Raptiva; Genentech), and rituximab (Rituxan/MabThera; Genentech, Biogen Idec) for the treatment of multiple sclerosis (MS), Crohn's disease (CD), severe forms of plaque psoriasis, hematologic malignancies, and rheumatoid arthritis (RA) [8].

Therefore, taking into account the important role of host immunity in PML pathogenesis, current understanding of the JCV infection and the new immunological and molecular findings relating to the PML pathogenesis have been comprehensively revised in this review.

## 2. JCV: Description of a Neurotropic Virus

JC virus has a capsid size of 45 nm with a naked icosahedral structure. Viral DNA is double-stranded, supercoiled, and of 5.13 Kb in length. The genome is transcripted on both strands and encodes for the early genes counterclockwise and for the late genes clockwise. The early proteins are the large T antigen (TAg), the small t antigen (tAg), and T′135, T′136, and T′165. Early proteins are nonstructural but multifunctional proteins encoded by five transcripts alternatively spliced from a viral early precursor mRNA. All these proteins are involved in the regulation of the virus cycle and in cell transformation. TAg is produced prior to viral replication, and it is a crucial DNA binding protein necessary for replication and transcription of viral DNA. In permissive cells, in fact, the JCV entry into the nucleus is followed by the transcription of the early gene TAg. On the other hand, TAg represses early genes transcription and stimulates viral genome replication and late genes transcription. Moreover, since viral replication also requires host cell proteins, such as DNA polymerase *α* and many transcription factors, TAg modulates cellular signaling pathways to induce quiescent cells to enter S phase in which cellular DNA is replicated. A key event in this process is the interaction of TAg with members of the retinoblastoma (Rb) protein family and p53, resulting in a consequent progression of the cell cycle [9]. Interaction with p53 also prevents apoptosis induced by checkpoint activation when cells aberrantly enter S phase [10]. Additionally, large T antigen can promote viral replication in G2-arrested cells by inducing DNA damage response pathways, and this function was related to binding of cellular DNA by TAg [9]. In nonpermissive cells, TAg has the ability to dysregulate several signaling pathways such as WNT/*β*-catenin that is responsible for the control of cell proliferation and IGF-IR/IRS-1 pathway involved in DNA repair fidelity [5].

Regarding T′ proteins, it seems that they influence cellular growth characteristics altering the phosphorylation status of cellular p107 and p130 proteins. They can be also found into the cellular nucleus and can be phosphorylated in a cell-cycle-dependent manner [11].

Small t antigen plays a significant role in the replication of viral DNA altering the activity of cellular protein phosphatase 2. In addition, it binds to the viral agnoprotein and to the Rb proteins (pRB, p107, and p130) influencing cell cycle progression [12].

Viral structural proteins are the three capsid proteins VP1, VP2, and VP3. In addition there is the late protein named agnoprotein. VP1 is responsible for the icosahedral structure of JCV and contains the epitopes for antibody induction and recognition. VP2 and VP3 are minor structural components and they are necessary for JCV propagation [7]. Agnoprotein directly interacts with TAg and contributes to the oncogenesis interfering with DNA repair, associating with several tumor suppressor proteins: the result is the uncontrolled cell proliferation [5]. Finally it has been demonstrated that agnoprotein acts as a viroporin [13].

**Figure 1:**
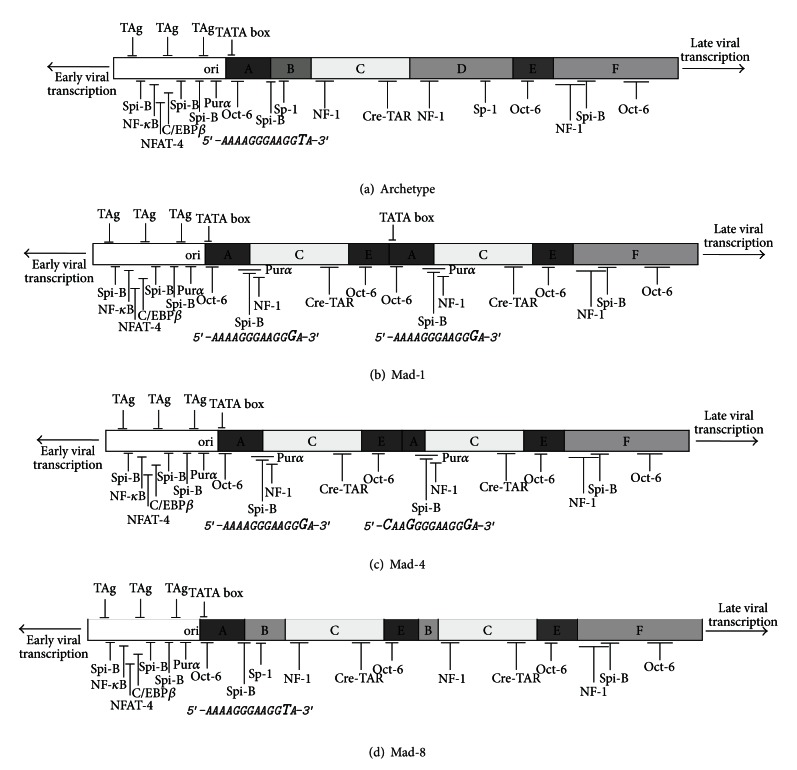
JCV noncoding control regions (NCCRs) containing cellular transcription factor-binding sites of archetype variant CY (a), commonly found in urine of healthy individuals and Mad-1 (b), Mad-4 (c), and Mad-8 (d) NCCR variants, illustrative of the major NCCR arrangements found in PML tissue.

The early and late genes are physically separated by the NCCR that is the most variable portion of the viral genome. The NCCR contains a bidirectional promoter and the viral origin of replication (*ori*). The archetype NCCR (CY strain) is divided into six regions named box A (36 bp), B (23 bp), C (55 bp), D (66 bp), E (18 bp), and F (69 bp) (Figure 1). Each region contains binding sites for cellular transcriptional factors involved in viral transcription. These binding sites undergo deletion and enhancement process that could generate variants with a different viral fitness in specific anatomical site [14, 15]. JCV archetype sequence, found in the kidney and urine, is not associated with PML and is not infectious in tissue culture models. Prototype NCCRs are variants isolated from tissues of patients with PML and are named on the hypothesis that the prototypes result from a rearrangement of the archetype sequence [16]. The original prototype is the Mad-1 isolate that contains a 98-bp tandem repeat (A-C-E-A-C-E-F) (Figure 1) [17]. Based on the occurrences of deletions and duplications, JCV isolates are assigned to two classes: the class I viruses characterized by the presence of the 98-bp tandem repeat within the NCCR (i.e., Mad-1) and the class II viruses containing strains that exhibit variations from the NCCR class I [18]. A particular NCCR structure is observed in HIV-positive subjects with PML: in fact this variant is characterized by multiple duplications of upstream Tat-responsive DNA element (up-TAR). Up-TAR is important for HIV-1 Tat stimulation of the JCV late promoter. Specifically, Tat enhances the ability of Pur-*α* to bind the up-TAR element and thus synergistically activates transcription [19, 20]. On this basis, it has been suggested that HIV Tat protein may contribute to the pathogenesis of PML (see below) (Figure 1) [13, 14].

## 3. PML Clinical Features

Clinical presentation of the PML is extremely varied: the most common symptoms include visual disturbances, behavioral alterations, and hemiparesis. Cognitive decline without accompanying deficits in motor or sensory function is uncommon [3]. Lesions are immediately adjacent to hemispheric cortical structures, and they contain enlarged oligodendrocytes and astrocytes with large and multiple nuclei. Initially, there are multiple foci of demyelination distributed in subcortical white matter; successively virus spreads from cell to cell and each focus grows. Microscopic areas of necrosis can become macroscopic plaque lesions [7]. Optic nerve involvement and fever are not features of PML and spinal cord disease is also rarely associated with disease progression [3].

Typically, PML is a confluent, bilateral but asymmetric, supratentorial white matter disease. However, it can be unilateral, and there may be a single lesion. Asymmetric multifocal bilateral confluent supratentorial lobar white matter involvement is the most common manifestation [21]. The parietal lobe is most commonly involved, followed by the frontal lobe. Supratentorial lesions typically involve subcortical white matter with a scalloped appearance [22]. White matter involvement has been reported to start in the subcortical regions, the site of highest blood flow, and then to move into the deep white matter in the centrum semiovale and periventricular regions [23]. Gray matter lesions are associated with white matter involvement in almost all cases. The thalamus is the most common area, followed by the basal ganglia. In the late stage of the disease, there is generalized atrophy and diffuse white matter involvement [21].

## 4. Link between Progressive Multifocal Leukoencephalopathy and Host Immunity Throughout the HAART Treatment and the New Immunomodulatory Drugs

### 4.1. Transmission and Immune Response against JC Virus

**Figure 2:**
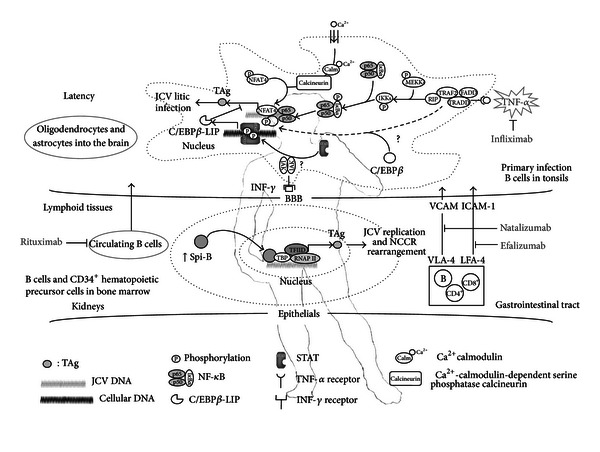
PML pathogenesis: a viral reactivation from a latent state rather than a primary infection. Role of immunomodulating drugs and host immune system.

JCV infection is widespread in the general population with more than 80% being seropositive by adulthood [24]. Nevertheless, the mode of transmission is not yet well defined although the presence of JCV DNA in B cells and stromal cells of the tonsils supports the proposal that tonsils may serve as an initial site of viral infection [25]. Virus might enter the upper respiratory tract by close interpersonal contact or via fomites and presumably spread by the hematogenous route from the primary site of infection to secondary sites such as kidneys, bone marrow, lymphoid tissues, and brain to establish focal areas of infection or persistence (Figure 2) [26]. Potential alternative modes of transmission include urinooral, transplancental, and transmission by blood transfusion, semen, and organ transplantation [27]. Archetype virus is also isolated from sewage samples from different geographical areas suggesting a possible transmission by contaminated food, water and fomites [28].

Upon the suppression of CD4^+^ and CD8^+^ T-cell mobilization, as occurs with HIV infection or during immune-modulating therapy (such as natalizumab, efalizumab, and rituximab), the JCV enters the brain, either within B cells or as cell-free virus, where it infects and kills oligodendrocytes, leading to demyelination. In particular the lack of surveillance, normally imposed by the immune system, could enhance the transcriptional activity of both NFAT4 and NF-*κ*B, that are under proinflammatory cytokine control and can also increase JC early genes (in particular TAg) transcription in response to TNF-*α* stimulation [29, 30]. Additionally, C/EBP*β* has been proposed to repress early transcription and is also under proinflammatory cytokine control [31]. As proposed for the inhibition of HIV transcription in monocyte-macrophages, in astrocytes the binding of IFN to its receptor may begin a JAK/STAT signaling cascade that results in increased production of C/EBP3-LIP, repressing the JCV early genes transcription. Therefore, interplay between the positive effects of NF-*κ*B and the negative effects of C/EBP*β* upon JCV genes transcription may be a key factor in the balance of JCV latency and reactivation. NF-*κ*B is an inducible transcription factor that regulates the expression of many cellular and viral genes. NF-*κ*B exists in cells as a hetero- or homodimer consisting of the Rel family of proteins which is comprised of RelA (p65), RelB and c-Rel, p50/p105 and p52/p100. These are normally complexed in the cytoplasm with the inhibitor protein I*κ*B. Upon stimulation by cytokines, upstream protein kinases are activated and I*κ*B becomes phosphorylated and targeted for ubiquitination and degradation. This releases NF-*κ*B to translocate to the nucleus where it activates gene expression [32] (Figure 2). Another family of transcription factors that are modulated by cytokines is comprised of the CAAT/enhancer binding proteins (C/EBPs). The C/EBP family contains six members (*α*, *β*, *γ*, *δ*, *ε*, and *ζ*), which contain a C-terminal DNA-binding domain, a leucine zipper domain that mediates homo- and heterodimerization, and an N-terminal transactivation domain. In particular, in addition to full-length (38 kDa) C/EBP*β*, two smaller forms of C/EBP*β* exist: liver-enriched transcriptional-activator protein (C/EBP*β*-LAP, 35 kDa) and liver-enriched transcriptional-inhibitory protein (C/EBP*β*-LIP, 20 kDa), which have common C-termini containing the leucine-zipper and DNA-binding domains but different N-termini resulting in changes to the transactivation domain. C/EBP proteins are regulated by cytokines and play important roles in many cellular processes. Direct physical and functional association can occur between members of NF-*κ*B and C/EBP proteins involving interaction of the Rel domain of NF-*κ*B with the bZIP domain of C/EBP [33]. Since both p65 and C/EBP*β* are regulated by signal transduction pathways activated by cytokines and immunomodulators, cross-communication between these two transcription factors may be important in controlling the balance of JCV latency and reactivation that occurs in response to immunosuppression. Moreover, the regulation of C/EBP*β* occurs at a number of levels, including gene transcription, translation initiation site selection, protein-protein interactions, and phosphorylation-dependent changes in DNA-binding activity, potential protein activation, and its subcellular localization [33, 34]. Therefore, the C/EBP*β* regulation pathways could also be implicated in the modulation of JCV transcription. Note, all three isoforms of C/EBP*β* (full length, LAP, and LIP) are expressed in human astrocytic and oligodendroglial cells, which are permissive for JCV replication. From these considerations, it was postulated that cytokines modulating NF-*κ*B and C/EBP*β* activities control JCV reactivation in the brain [35], and that latent virus in oligodendrocytes and astrocytes can be activated by proinflammatory cytokines allowing expression of viral proteins and viral replication. In highly immunosuppressed individuals, the virus may then be able to infect neighboring cells leading to the spread of virus, because of the lack of an adequate antiviral immune response, and leading to the development of a PML lesion. In particular, the inhibition of CTL migration operated by natalizumab could enhance the spread of virus. On the contrary, few cases are associated with the TNF-*α* blocking although anti-TNF-*α* medications would trigger demyelination. It has been hypothesized that exposure to anti-TNF-*α* might, between other effects, increase survival of autoreactive peripheral T cells penetrating the CNS, produce proinflammatory cytokines such as IFN-*γ*, and cause demyelination [36] (Figure 2). In this scenario, a TNF-*α* blocking could unbalance the fine interaction between NF-*κ*B and C/EBP*β* activities, encouraging a JCV latent state. However, since patients treated with anti-TNF-*α* drugs develop different forms of CNS and peripheral nervous system demyelination, adalimumab and infliximab are recognized treatments for RA, psoriatic arthritis, ankylosing spondylitis, and CD, but not for MS, a demyelinating autoimmune disease treated with natalizumab. The nuclear factor of activated T cells (NFAT) is another transcription factor family under proinflammatory cytokine control. In particular, NFAT is the primary target of Ca^2+^-calmodulin-dependent serine phosphatase calcineurin, a crucial component of the calcium-signaling pathway that can stimulate the production of inflammatory cytokines in response to extracellular stimuli [37]. Treatment of glial cells with an inhibitor of the NFAT family also inhibited JCV infection, implicating NFAT involvement in the life cycle of JCV. B cells may play an important role in the pathogenesis of PML, in addition to be a potential site of viral latency. Since it has been suggested that the viral genome rearranges during DNA replication; an attractive model is that these events occur in B cells, since these cells possess the Rag1 and Rag2 enzymes for immunoglobulin gene rearrangements. NCCR recombination may lead to acquisition of transcription factor binding sites that are important for pathogenesis. A recent example was described in patients receiving infliximab, where an archetype-like NCCR contained sequences that led to TATA box-associated Spi-B sites known to be important for viral replication, while JCV in the urine contained an archetype NCCR sequence [38]. Additionally, evidence that changes in transcription factors can affect viral transcription could be found in the observation that natalizumab treatment upregulates factors involved in B cell differentiation, including Spi-B [39]. Spi-B binding sites in the promoter/enhancer of JCV variants are located directly adjacent to TATA boxes, that are essential for transcription of early and late viral genes. Spi-B is a transcription factor that can cooperate with pRB and TATA-binding protein (TBP) to alter expression of proteins involved in B cell maturation [40, 41]. TBP binds TATA box elements in promoters and it is a subunit of the basal transcription complex TFIID, which increases RNA polymerase II activity. Recruitment of the TFIID complex to JC viral promoters by Spi-B and TBP is an attractive model for the activation of JCV gene expression (Figure 2).

JCV is a neurotropic virus; nevertheless, it is still incompletely understood how the virus infects the central nervous system (CNS). There are two possibilities: JCV infects the CNS in case of alteration of the immune response; alternatively, the virus infects the CNS and persists there for many years in immunocompetent individual. When alteration of the immune system occurs, viral infection emerges [7]. Within the brain, JCV can infect both oligodendrocytes and astrocytes [42]; occasionally JCV infects the cerebellar granule cells [3]. Immune control of JC virus is based mainly on cellular immune response. Cytotoxic CD8^+^ T lymphocytes (CTLs) recognize the epitopes of viral proteins presented on the class I HLA molecules preventing further spread of the virus. CTLs are usually detected in the blood of PML survivors, in PML lesions where they aggregate around infected cells and rarely in patients with PML, who have a fatal outcome within 1 year from disease onset [43]. Specific CD4^+^ T cells have been detected in the blood of patients who have survived PML, and the number of these cells correlates with JC virus clearance from the cerebrospinal fluid (CSF) [6].

The humoral immune response against the JC virus has been extensively studied. The first test to estimate seroprevalence was the haemagglutination inhibition assay based on the ability of JCV antibodies in the serum to prevent agglutination of human type O erythrocytes [44]. Nowadays the use of different technologies in the tested populations (i.e., quantitative enzyme immunoassay on recombinant VP1 protein) revealed a great variability of viral seroprevalence in adults and children. It might be explained on the basis that primary infection is not associated with a recognizable clinical event. Eleven JCV genotypes have been found to be serologically distinct and there is no clearly defined JC virus seronegative population [45].

Regarding HIV positive patients, although intrathecal JC virus antibody becomes detectable with JC viral clearance after treatment with highly active antiretroviral therapy (HAART), neither the presence of intrathecal nor serum specific antibodies prevent the onset of PML [46].

### 4.2. PML and HIV/AIDS in the Era of HAART

PML was first described as a complication of chemotherapy in hematological patients, but from 1980s it emerged as a major complication of HIV infection and its incidence increased 50-fold: now PML affects nearly 1 in 200,000 people [3]. PML is the cause of death in 3 to 5% of AIDS patients, but with the introduction of HAART this mortality rate is expected to decrease [8]. This rate of disease may be due to several factors, including duration and extent of immunosuppression, changes in cytokine secretion induced by HIV, viral interactions in coinfected cells, and increased blood-brain barrier (BBB) permeability allowing for B cells infected by JCV to enter the brain [47]. CD8^+^ T cell responses specific to JCV are important to control JCV [43, 48, 49], and, during chronic viral infections, CD4^+^ T cells are required to maintain a CD8^+^ T cell response [50]. Moreover increased circulation of B cells, which may favor JCV crossing of the BBB, has also been observed during HIV infection [51]. Thus, HIV infection seems to promote an immunological state that favors the onset of PML. Additionally, studies have shown a potential synergistic role of HIV and JCV at the molecular level, an effect that is likely a cause of the high rate of PML in HIV-infected individuals. Interestingly, HIV and JCV may share an immune cell site of latency, as both JCV and HIV have been reported to be present in CD34^+^ bone marrow progenitor cells and may be reactivated upon differentiation to B lymphocytes [52, 53]. Focusing on molecular pathways of interaction between viruses and host cell, the HIV Tat protein has been shown to increase transcription from JCV [54–56], and this result has been confirmed by a recent case report on a HIV-associated PML patient with two variant of JCV detected in the CSF, an archetype variant and an archetype-related variant with a duplication of the cre-TAR binding site for the HIV Tat protein (Iannetta and colleagues unpublished data). Moreover, archetype JCV, which normally cannot be efficiently propagated in cell culture, has been found to replicate in cells expressing HIV Tat [55]. The viruses also share requirements for transcription factors, including members of the NF-1 family [57], and both viruses interact with the cellular protein Pur-*α* [58]. Interestingly, the interaction between HIV Tat and cellular Pur-*α* has been shown to play a role in DNA repair [59], which could potentially cause increased JCV rearrangements in coinfected cells, leading to an increased chance of JCV NCCR sequences associated with PML. HIV infection of the brain also causes upregulation of cytokines which attract lymphocytes, as well as an increase in cell adhesion molecules which may facilitate crossing of JCV-infected cells [53, 60].

The HAART treatment in HIV patients is the sole solution to manage the onset and/or the progression of the PML in those patients. However the immune reconstitution operated by HAART in AIDS-associated PML carries with it the potential for severe and life-threatening side effect from immune reconstitution inflammatory syndrome (IRIS) [61]. IRIS is due to an immune reconstitution which leads to infiltration of lymphocytes into the PML lesions. In particular, IRIS is thought to result from resumption of immune surveillance in the CNS and might be associated with an initial worsening of neurological symptoms. IRIS probably corresponds to CD8^+^-mediated inflammatory changes and can often be identified by gadolinium enhancement on magnetic resonance (MR) [62]. In fact, clinical diagnosis is based on MR and an increase of the CD8^+^ and CD4^+^ T cell counts, which also represents the most predictive factors for survival from PML (counts over 200) [63]. The diagnostic utility of CSF JCV DNA for distinguishing between IRIS PML and PML progression is unknown and requires further study.

More study is needed to determine the functional interplay between JCV and HIV, but it is clear that there is significant interaction between the viruses at the molecular, cellular, and immunological levels [53].

### 4.3. Immunomodulatory Therapies and PML

The PML onset after using monoclonal antibodies (mAbs) therapies has not only raised concerns about the safety profile of these agents, but also provided a window into the pathogenesis of PML.

The mAbs that target (1) cell adhesion molecules, such as very late antigen-4 (VLA-4) (natalizumab for relapsing-remitting forms of MS and CD) or lymphocyte function-associated antigen-1 (LFA-1) (efalizumab for severe forms of plaque psoriasis) to prevent extravasation of inflammatory T cells into tissues, or (2) the cell surface marker CD20 (rituximab for hematologic malignancies and RA) to deplete peripheral circulating B cells, have all been associated with PML. Thus, these mAbs currently carry US Food and Drug Administration (FDA)-mandated “blackbox” warnings [64]. Furthermore, antitumor necrosis factor alpha (anti-TNF-*α*) agents, such as adalimumab (Humira; Abbott) and infliximab (Remicade; Centocor Ortho Biotech), are used in several autoimmune diseases, mainly in CD and severe forms of plaque psoriasis. Several cases of demyelinating events of the nervous system have been reported although the incidence of such events is relatively low [8, 65].

Natalizumab is a humanized mAb which binds to the *α*4 chain of *α*4*β*1 (or VLA-4) and *α*4*β*7 integrins on the hematopoietic cells. Binding of natalizumab to *α*4*β*1 integrins prevents adhesion to the vascular cell adhesion molecule (VCAM) and thus the transmigration of activated lymphocytes through the BBB. Multiple sclerosis is characterized by chronic leukocyte infiltration into the brain, and natalizumab blocks this infiltration by preventing extravasation through cell adhesion molecule binding (Figure 2). As the blockade of *α*4*β*7 integrins prevents the adhesion of activated T lymphocytes to the mucosal addressin cell adhesion molecule 1 (MAdCAM-1) and their extravasation into the gastrointestinal mucosa [53], natalizumab has been also approved for the treatment of moderate-to-severe CD in the USA [66]. Unfortunately, the efficacy of natalizumab was overshadowed by the occurrence of PML in two MS patients and one CD patient [4]. Thus, in 2005, Biogen Idec (Cambridge, MA, USA) and Elan (Gainesville, GA, USA) voluntarily suspended their marketing. After a large assessment of clinical, MRI, and laboratory data of patients treated with natalizumab, the US FDA and the European Medicines Agency (EMEA) reintroduced its market with a close postmarketing surveillance. Among 82,732 patients treated with natalizumab worldwide until to 2011, 102 cases of PML have been reported, and this number seems expected to rise. The risk of PML increases with duration of exposure to natalizumab over the first 3 years of treatment, and median treatment duration to onset of symptoms was 25 months (range, 6–80 months). To date, the true incidence of PML due to current immunomodulatory therapies remains to be determined, but it has been estimated to be approximately 3.85 per 1,000 patients treated with more than 24 infusions [4, 53].

Since natalizumab has not been consistently associated with opportunistic infections other than PML, there must be a specific mechanism that causes PML mainly in patients affected by autoimmune diseases. By binding to integrins on CD34^+^ hematopoietic precursor cells, natalizumab treatment results in an increase in CD34^+^ cells in both the bone marrow and the blood [67]. Natalizumab also increases circulating pre-B and B cells in the periphery and prevents homing of CD34^+^ progenitor cells to the bone marrow and of pre-B cells to lymph node marginal zones [8, 39, 68]. Natalizumab treatment also results in an increase of factors involved in B cell differentiation, including Spi-B, in the peripheral blood [39]. As Spi-B has been shown to increase JCV transcription, this may be a mechanism for the high risk of PML in those treated with natalizumab (see below) [69]. This dynamic creates a favorable environment for JC virus, which can reside in a latent state in the bone marrow for long periods before the development of PML and which can use B cells and their DNA-binding proteins to initiate viral replication. However, Warnke and colleagues support the hypothesis that CD34^+^ progenitor cells mobilized by natalizumab are not a relevant reservoir for JC virus [70].

Interestingly, JCV-specific T cell response seems to increase in natalizumab-treated patients. Nevertheless, by preventing autoimmune T cells from reaching the brain, natalizumab may also impair the immune surveillance against foreign antigens such as JCV [71]. In fact, natalizumab decreases dramatically the number of dendritic and CD4^+^ T cells in the cerebral perivascular space, as well as B cells and T cells in CSF. Thus, how would JCV reach the brain if precisely the BBB is blocked? One possibility is that JCV is already present in the brain of some individuals before natalizumab treatment: in fact, Perez-Liz and colleagues have detected JCV DNA fragments in normal brain tissue [35]. Moreover, JCV could also use some infected B lymphocytes and CD34^+^ hematopoietic precursor cells to enter the CNS and to infect glial cells. Nearly all patients with MS, who develop PML following treatment with natalizumab, develop also IRIS, which carries a high morbidity and mortality rate. The rapid restoration of T cells' capacity to cross the BBB, following the suspension of natalizumab treatment, can lead to a massive infiltration of PML lesions by JCV-specific CTL. Although the most important factor for a favorable outcome of PML is immune reconstitution, in the case of IRIS, there can be a transient worsening of the symptoms due to the massive inflammation. This syndrome seems to be more common and more severe in patients with natalizumab-associated PML than in patients with HIV-associated PML. Management of PML has routinely used plasma exchange or immunoabsorption to hasten clearance of natalizumab and shorten the period in which natalizumab remains active (usually several months) [72, 73].

Rituximab is an anti-CD20 humanized monoclonal antibody that fixes complement. Binding of CD20, expressed on B cells, results in downregulation of the B cell receptor and cytolytic apoptosis of CD20^+^ B cells. The administration of rituximab results in depletion of CD20^+^ B cells in the peripheral blood and CSF (Figure 2) [8]. It is approved for CD20^+^ B cell non-Hodgkin's lymphoma, untreated chronic lymphocytic leukemia (approved by EMEA in 2009), and as a second-line treatment for rheumatic arthritis. To date, 114 PML cases have been associated to treatment with rituximab, always used in combination with other immunosuppressive treatments [64, 65]. Its mode of action may be based on the decrease of both the humoral and cellular immune responses, due to diminished help provided by B cells to T cells [74]. However, despite of these cases of PML emerged after treatment with rituximab, the pathophysiology of PML associated with this mAb remains uncertain. Rituximab treatment does not result in T cell depletion, as seen in AIDS. However, as with natalizumab, pre-B and B cells may be mobilized from the bone marrow and lymph nodes to replace depleted CD20^+^ B cells in the periphery, and there is an association with higher levels of CD34^+^ progenitors in the periphery [8]. Thus, it is possible that the subsequent replacement of the mature B cell population results in expansion of pre-B-cell-harboring latent JC virus that can use these lymphocytes to reach the CNS and to infect permissive cells, such as oligodendrocytes. For the time being, in Europe and in the USA, the manufacturer in collaboration with the respective health agencies proposes a patient alert card mentioning the risk of PML for patients treated with rituximab [64].

Efalizumab is a humanized IgG1 monoclonal antibody targeting CD11*α*, a subunit of the leukocyte function-associated antigen type 1 (LFA-1), a T lymphocyte adhesion molecule. LFA-1 binds intercellular adhesion molecule 1 (ICAM-1), which allows migration of T lymphocytes from circulation into sites of inflammation (Figure 2). Efalizumab also downmodulates expression of VLA-4 and results in T cell hyporesponsiveness [75]. It was used for the treatment of moderate-to-severe plaque psoriasis and was withdrawn from the market in April 2009 because of the occurrence of PML at an incidence of approximately 1 in 500 [53]. By binding to the domain I of the *α* chain of CD11*α*, it also triggers a conformational change in LFA-1 and can affect apoptosis, cytotoxicity, cell proliferation, cytokine production, antigen presentation, and gene activation of T lymphocyte. This set of events affects psoriasis pathogenesis at multiple levels, perhaps most importantly by inhibiting the initial T cell activation in lymph nodes, preventing binding of T cells to endothelial cells and blocking trafficking of T cells from the circulation into the psoriatic skin, preventing their reactivation in the dermal and epidermal layers [76]. Blockade of costimulatory molecules on T cells, particularly CD11*α*, as occurs with efalizumab, also results in a sustained unresponsiveness to viral and other pathogens in animal models. It has been also demonstrated to reduce T cell activation produced by polyclonal stimuli; this T cell hyporesponsiveness is fully reversible following efalizumab washout [77]. Although natalizumab and efalizumab increase peripheral blood leukocytes during treatment, CD8^+^ T cells are predominantly represented. Moreover, efalizumab reduces cutaneous dendritic cells, but its effect on cerebral perivascular dendritic cells is unknown [78]. Interestingly, as natalizumab, efalizumab is directed against members of the integrin family, which raises the question whether there may be a relationship between antiintegrin agents and JCV/PML.

The anti-TNF-*α* biological agents, such as adalimumab and infliximab, are widely used in several autoimmune diseases, mainly in CD and severe forms of plaque psoriasis. Several cases of demyelinating events of the nervous system in association with the anti-TNF-*α* therapy have been reported, prompting a heightened surveillance of treated patients, although the incidence of such events is relatively low. Since patients treated with anti-TNF-*α* drugs develop different forms of CNS and peripheral nervous system demyelination, adalimumab and infliximab are recognized treatments for RA, psoriatic arthritis, ankylosing spondylitis, and CD, but not for MS [65]. The mechanism by which anti-TNF-*α* medications would trigger demyelination remains unexplained. It has been hypothesized that exposure to anti-TNF-*α* might, between other effects, increase survival of autoreactive peripheral T cells penetrating the CNS, produce proinflammatory cytokines such as interferon gamma (IFN-*γ*), and cause demyelination [36]. However, recently, the first case of PML in a man with erosive RA after 3 years of treatment with infliximab [79] has been reported. The TNF-*α* blocking could also result in decreased expression of interleukin (IL)-6 and IL-1, IFN-*γ*, and other proinflammatory cytokines, with a subsequent reduction in inflammation at the macrophage site of TNF-*α* production (Figure 2). A reduction in cell recruitment occurs also through reduced expression of CD3^+^, CD68^+^, VCAM-1, ICAM, and E-selectin. A reduction in T cell activity has been theorized to occur through a reduction in IFN-*γ*, STAT-1, granzyme B, and T cell inflammatory gene expression as well as a reduction in dendritic cell-mediated T cell activation [80]. Infliximab also induces apoptosis in TNF-*α* producing T cells [81]. Therefore, although infliximab reduced clinical symptoms, it also could unbalance the local immune surveillance, particularly the T cell activation and the consequential reduction of IFN-*γ* expression, involved in the antiviral state control, and enhance the latent JCV reactivation, with direct implications on the risk of PML [82]. Nevertheless, because of the concern that anti-TNF-*α* treatment may trigger or worsen demyelination in some patients, a baseline brain MR is recommended prior to the initiation of anti-TNF-*α* treatment [83].

In conclusion, it is likely that all these therapies could lead to PML due to a decrease in immune surveillance. Conversely, several of these therapies, notably natalizumab and rituximab, result in a decrease of mature B cells in the periphery and a subsequent mobilization of immature B cells from the bone marrow, potentially disseminating latent virus to the brain. Recombination of DNA in B cells also offers an attractive model for the changes in the viral NCCR that are necessary to increase pathogenicity and replicative efficiency of the virus in glial cells (see below) [4, 53].

## 5. PML Pathogenesis from Early Viral Genes Transcription to Oligodendrocytes Lysis

The JCV life cycle starts with viral early gene transcription (I step), mainly TAg, and several host factors are required and may contribute to this process. In the presence of TAg, the host DNA polymerase, and a number of other cellular proteins participate in JCV DNA replication (II step). However, the kinetics of JCV replication is a slow process: in fact, even in susceptible cells in which TAg is already present, DNA replication is detectable only after several days [84]. Moreover, the viral gene transcription has been shown to be regulated by cell-specific factors, while the viral DNA replication is most likely regulated by species-specific factors. These species-specific factors, which may be one or more components of the DNA polymerase, allow JCV DNA replication only in primates [53, 85]. Therefore, it is clear that viral DNA replication proceeds when TAg accumulates. TAg binds preferentially to a site located in the viral DNA *ori*, closest to first TATA box in the NCCR. When TAg binds JCV DNA, it promotes the YB-1/Pur-*α* switch to viral late gene transcription. TAg also interacts with host cell DNA replication machinery to directly initiate replication (Figure 1) [53]. Several cellular proteins have been implicated in directly increasing viral DNA replication. In particular, the NF-1 family has been extensively shown to modulate JCV replication and viral genes expression *in vivo* [86].

NF-1 is a family of transcription factors that contains four members—A, B, C, and X— and each of them can activate or repress transcription through several mechanisms. The isoform NF-1A, which is expressed in several JCV nonpermissive cell types, has been shown to decrease viral late protein expression [87], while NF-1X increases viral gene expression and is highly expressed in cells permissive to JCV replication [57]. Therefore, the NF-1 sites in the tandem repeat enhancer of JCV NCCR could be possible determinants of glial cell specificity during JCV infection. However, NF-1 binds JCV genome in a variety of cell types, suggesting that NF-1 activity is not restricted to the brain and could be involved in the basal activity of the JCV promoters [86]. In particular, NF-1X is overexpressed in the brain where it binds the JCV NCCR and affects both early and late viral transcription [88]. Moreover, the NF-1A isoform, recently reported as a negative regulator of JCV activity, is expressed at higher levels than NF-IX in hematopoietic progenitor cells [57, 87]. In these cells the JCV genes expression is minimal suggesting that NF-1A may contribute to repression of viral activity in nonsusceptible cells or cells where the virus is in a latent state. These results demonstrate that the NF-1 protein family has antagonistic effects on JCV gene expression in both the immune system and in the brain [16].

Another aspect to consider is that the repeated sequence of the NCCR enhances its own recombination during viral DNA replication. This recombination can explain the large variety of sequences of NCCRs derived from PML patients. The prototype Mad-1 NCCR contains two identical tandem repeats (two sets of A-C-E sequence) followed by the box F. Most PML-derived NCCR sequences contain some version of these repeats, with deletions and insertions in some cases. Archetype CY, which is found primarily in the urine of healthy subjects, does not contain repeats and generally has a NCCR consisting of an A-B-C-D-E-F sequence (Figure 1). One hypothesis for viral transmission and evolution is that archetype-like virus is the circulating form and that deletions (generally of B and D box) then occur, followed by duplication of remaining sequence. This then leads to a pathogenic form of the virus able to replicate efficiently in glial cells [47]. Moreover, there is evidence that the archetype “d” region may be inhibitory to JCV growth in some cells: so its deletion allows productive infection of other cells [89]. Recombination of the JCV NCCR may also be explained by its interaction with cells of the immune system (see below).

JCV early genes transcription occurs in absence of *de novo* protein synthesis and utilizes only host proteins. Moreover, the regulation of transcription is dependent on the sequence of the NCCR, as well as the availability of host transcription factors. Mad-1 NCCR transcription factor binding sites include four Oct-6/tst-1/SCIP binding sites [90], two Pur-*α* binding sites, two YB-1 binding sites [91], four NF-1 binding sites [87, 92], and six Spi-B binding sites [69]. The Mad-1 NCCR is composed of two 98-bp tandem repeats, each containing a TATA box (Figure 1). TATA boxes were duplicated in Mad-1 because they were functional for early transcription, but they may vary in importance depending on the cell type infected. Recently, it has been shown that the Spi-B transcription factor binding sites in the second tandem repeat can compensate the loss of the second TATA box [69]. However, NCCR variants lacking repeat sequences show greatly reduced early transcriptional activity in comparison to both Mad-1 and Mad-4 (Figure 1) [89].

Changes in NCCR result in changes in cellular transcription factor binding sites, which affect tissue specificity and activity of viral transcription and replication. The JCV NCCR contains a bidirectional promoter and the viral origin of replication (*ori*). The genome is transcripted on both strands and encodes for the early genes counterclockwise (*Early viral transcription*) and for the late genes clockwise (*Late viral transcription*). The archetype NCCR is divided into six regions named box A (36 bp), B (23 bp), C (55 bp), D (66 bp), E (18 bp), and F (69 bp) [93]. JCV archetype sequence is not associated with PML and is not infectious in tissue culture models. Rearranged NCCRs are variants isolated from tissues of patients with PML. The original prototype is the Mad-1 isolate that contains a 98-bp tandem repeat (A-C-E-A-C-E and F) [17]. Archetype CY does not contain repeats as well as in the PML-associated rearranged NCCR. One hypothesis for viral transmission and evolution is that archetype-like virus is the circulating form and that deletions (generally of B and D box) then occur, followed by duplication of remaining sequence. This then leads to a pathogenic form of the virus able to replicate efficiently in glial cells. A particular NCCR structure is observed in HIV-positive subjects with PML: in fact this variant is characterized by multiple duplications of upstream Tat-responsive DNA element (*Cre-TAR*). *Cre-TAR* is important for HIV-1 Tat stimulation of the JCV late promoter. On this basis, it has been suggested that HIV Tat protein may contribute to the pathogenesis of PML. Moreover, in the JCV NCCR of HIV-negative subjects with PML, Mischitelli and colleague [15] have also found enhancements of both Sp-1 binding sites in box B and box D. Sp-1 is a cell transcriptional factor, which carries out two functions in JCV replication, the first one is the activation of JCV early promoter in both glial and nonglial cells in relation to its binding to box B site, and the second one is the TAg-mediated transactivation of viral late genes in relation to its binding to the site located in box D. Moreover, it is important to note that viral DNA replication proceeds as the early protein large T antigen accumulates. Large T antigen binds preferentially to a site (*TAg*) located in the viral DNA *ori*, closest to first *TATA box* in the NCCR. When large T antigen binds JCV NCCR, it promotes the Pur-*α*/YB-1 switch to viral late gene transcription. Large T antigen also interacts with host cell DNA replication machinery to directly initiate replication. Several cellular proteins has been implicated in directly increasing viral DNA replication. In particular, the NF-1 family of proteins have been extensively shown to modulate JCV replication and viral genes expression *in vivo*. NF-1 is a family of transcription factors that contains four members: A, B, C, and X. The isoform NF-1A, which is expressed in several JCV nonpermissive cell types, has been shown to decrease viral late protein expression, while NF-1X increases viral gene expression and is highly expressed in cells permissive to JCV replication. Therefore, the regulation of transcription is dependent on the sequence of the NCCR, as well as the availability of host transcription factors.

Mad-1 NCCR transcription factor binding sites generally include four Oct-6/tst-1/SCIP binding sites, two Pur-*α*/YB-1 binding sites, three NF-1 binding sites, and six Spi-B binding sites, three of which upstream the *ori*. The Mad-1 NCCR also contains two *TATA boxes *(B). The proximity of *TAg*, *TATA box*, and Spi-B-binding sites on the JCV NCCR *ori* region suggests that *TAg* and Spi-B may cooperate in recruitment of transcriptional apparatus components in immune cells, similar to the cooperation of *TAg* and Oct-6/tst-1/SCIP in glial cells. In particular, the NCCR sequence containing the inactive archetype Spi-B site (5′-AAAAGGGAAGGTA-3′) is conserved among viruses isolated from the urine of healthy individuals. However, the novel observation of one such point mutation that creates an active Spi-B-binding site (5′-AAAAGGGAAGGGA-3′) directly adjacent to the archetype *TATA box*, which converts archetype to a biologically active virus, further supports the hypothesis that a NCCR recombination toward more virulent variants occurs in the cells harboring the virus in a latent state. Importantly, similar mutations that create active Spi-B-binding sites adjacent to the TATA boxes of archetype-like viruses have been described in the brain of a patient with cerebellar atrophy and in intestinal biopsies of Crohn's disease patients treated with infliximab, suggesting that this type of mutation is supported during dissemination in the host. Finally, the JCV NCCR contains binding sites for a number of transcriptional enhancers and repressors. The proteins NF-1X, NFAT4, NF-*κ*B, and Spi-B have all been proposed to bind to all variants of the JCV NCCR and activate early viral genes transcription in various cell types, while NF-1A and C/EBP have been proposed to repress early viral genes transcription. Thus, it is likely that the activation of JCV is controlled by access to DNA binding proteins available in cells of the nervous system. In particular, Romagnoli and colleagues [31] show that the binding sites for C/EBP*β* (in particular C/EBP*β*-LIP) and NF/*κ*B-p65 in the JCV NCCR are adjacent but not identical. The C/EBP*β*-LIP binding site lies slightly closer to the JCV *ori* than the NF/*κ*B-p65 binding site. Surprisingly, mutations that removed either the NF/*κ*B-p65 or C/EBP*β*-LIP binding sites diminished basal promoter activity and also removed the ability of the promoter to respond to either transcription factor. This may be because mutations in this region (*ori* region in all four variants reported) are not well tolerated, and indeed this NCCR region is conserved between almost all isolates of JCV. It is also possible that the mutations in this region might affect the binding of other factors, and, for example, Manley et al. [29] have suggested that NFAT4 binds to this site (Figure 1).

Host transcription factor availability is the determining factor for early viral genes transcription, as well as the quantity of TAg produced. Although NCCRs containing repeats (such as Mad-1 or Mad-4) and variants isolated from PML strains have greater transcriptional activity [89], both the archetype and various PML isolates show increased transcriptional activity in glial cells rather than cells of nonglial origin. Once TAg is present, the difference in replication fitness between different variants of JCV becomes much less apparent [94]. Therefore, the ability of the virus to transcribe the early region of its genome is a major determinant of cell type specificity of the virus, and for these reasons much of the research has been focused on viral transcription. The JCV NCCR contains binding sites for a number of transcription factors and transcriptional repressors. The proteins NF-1X [57, 95], NFAT4 [29], NF-*κ*B [96], and Spi-B [69] have all been proposed to bind to variants of the JCV NCCR and activate early viral genes transcription in various cell types, while NF-1A [87] and C/EBP [31] have been proposed to repress early viral genes transcription (Figure 1). Thus, it is likely that the activation of JCV is controlled by access to DNA binding proteins available in cells of the nervous system.

The last step of JCV life cycle is the late viral genes transcription with the same transcription factors involved in the transcription of the early genes. In fact, by the interaction with TAg, NF-1 family members have been shown in several studies to increase early and late gene expression in a TAg-dependent manner [86, 97]. This system may be similar to a better-characterized switch in the JCV life cycle: in fact, the cellular proteins YB-1 and Pur-*α* interact with the viral TAg to regulate the switch from early to late gene expression [91, 98, 99]. Pur-*α* is also a strong activator of early genes expression [91], and, as TAg accumulates, it facilitates binding of YB-1 to the viral lytic control element (LCE). YB-1 and TAg stimulate late gene expression [91, 98], and thus Pur-*α*, YB-1, and TAg work as a genetic switch to shift gene expression from early to late viral genes.

Activated late gene expression requires TAg and occurs concurrently with DNA replication (229). TAg promotes late transcription by interacting with components of the basal transcription machinery, including TATA binding protein (TBP), TBP-associated factors (TAFs), and transcription factors [100]. Moreover, a number of DNA-binding proteins have been implicated in regulation of late viral transcription. In particular, NFAT4 has been shown to activate late-gene transcription, while C/EBP*β* appears to function as transcriptional repressor. Moreover, both NFAT4 and C/EBP*β* expressions are under proinflammatory cytokine control, such as TNF-*α* [29, 31]. Additionally, subunits of NF-*κ*B have been shown to increase late-gene expression and can also increase viral expression in response to TNF-*α* stimulation [30].

### 5.1. Immune Surveillance in the Central Nervous System and Immune-Regulated Cellular Pathway Implicated in Oligodendrocytes Lysis by JCV

Into the CNS, one of the main roles of microglia is to be the first line defense against infectious agents and injury-related products in the CNS parenchyma. Nonactivated microglia, particularly in the white matter, constitutively express low levels of HLA-DR in the healthy human brain. Indeed, the molecular markers of antigen presentation and activation, such as MHC II, CD80, CD86, CD40, and CD11a, are rapidly increased on microglia in response to pathological changes in the CNS, and these cells are able of presenting antigens to, and activating, T cells [101]. Specifically, the upregulation of MHC I by microglia is postulated to reflect a vigilant state of these cells, enabling them to present antigen to and engage CD8^+^ T cells early after infection [102]. Also, competent presentation of antigen to CD4^+^ T cells through MHC I and II on microglia during Theiler's murine encephalitis virus infection likely reflects an effort to clear the virus [103]. In addition to antigen presentation, microglia have all the machinery necessary to detect most microbes that access the CNS parenchyma and can rapidly mount a potent inflammatory response. Indeed, intraventricular administration of lipopolysaccharide rapidly induces a macrophage-like response with the release of cytokines (TNF-*α*, IL-1, interferons (IFNs), and others) and the production of many chemokines that alert and recruit more immune cells to the brain [104]. Although present in small numbers relative to peripheral organs, peripherally derived T cells, macrophages, and dendritic cells constitute another group of sentinels for CNS. Whereas microglia are the primary watchmen in the parenchyma of the brain and spinal cord, peripheral immune cells are recruited in specialized CNS compartments located outside the parenchyma. Immune cells could gain access to the CNS via the nonfenestrated vascularized stroma of the blood-CSF barrier that is surrounded by the choroid plexus epithelial cells, and the postcapillary venules that enter the parenchyma directly [105]. In all of these possible sites of extravasation, migration across the vascular wall and the glial limitans must occur for the cells to gain access to the parenchyma. Approximately 80% of immune cells found in the CSF of healthy individuals are T cells that have probably entered the CNS through the choroid plexus and meninges. As expected, the highest numbers of immune cells are located in brain areas where the tight junction barrier of the BBB is reduced. Not all studies agree with a population of CD4^+^ T cells in the healthy human brain. At least two separate groups have instead been identified: CD3^+^CD8^+^granzyme B^−^perforin^−^ lymphocytes and, to a limited extent, CD20^+^ B cells as the main cellular components [106]. Peripherally activated T cells gain essential surface molecules necessary to traverse the BBB into the CNS parenchyma. Capture and adherence of activated Th1 T cells to CNS venules were demonstrated to occur via interactions between VLA-4 on TH1 cells and VCAM-1 on endothelial cells, whereas diapedesis of TH1 cells across the venule wall is facilitated by leukocyte function-associated antigen (LFA)-1 [107].

Taking into account this scenario, upon the suppression of CD4^+^ and CD8^+^ T cell mobilization, as occurs with HIV infection, during chemotherapy or immunosuppressive therapy, the JCV enters the brain, either within B cells or as cell-free virus, where it infects and kills oligodendrocytes, leading to demyelination (Figure 2) (see above). In particular, from a molecular point of view, the lack of surveillance, normally imposed by the immune system, could enhance the transcriptional activity of both NFAT4 and NF-*κ*B, that are under proinflammatory cytokine control and can also increase JC early genes transcription in response to TNF-*α* stimulation [29, 30]. Additionally, C/EBP*β* has been proposed to repress early transcription and is also under proinflammatory cytokine control [31]. Therefore, interplay between the positive effects of NF-*κ*B and the negative effects of C/EBP*β* upon JCV genes transcription may be a key factor in the balance of JCV latency and reactivation (Figure 2).

NF-*κ*B is an inducible transcription factor that regulates the expression of many cellular and viral genes. NF-*κ*B exists in cells as a hetero- or homodimer consisting of the Rel family of proteins which is comprised of RelA (p65), RelB and c-Rel, p50/p105 and p52/p100. These are normally complexed in the cytoplasm with the inhibitor protein I*κ*B. Upon stimulation by cytokines, upstream protein kinases are activated and I*κ*B becomes phosphorylated and targeted for ubiquitination and degradation. This releases NF-*κ*B to translocate to the nucleus where it activates gene expression [32]. Another family of transcription factors that are modulated by cytokines is comprised of the CAAT/enhancer binding proteins (C/EBPs). The C/EBP family contains six members (*α*, *β*, *γ*, *δ*, *ε*, and *ζ*), which contain a C-terminal DNA-binding domain, a leucine zipper domain that mediates homo- and heterodimerization, and an N-terminal transactivation domain. In particular, in addition to full-length (38 kDa) C/EBP*β*, two smaller forms of C/EBP*β* exist, liver-enriched transcriptional-activator protein (C/EBP*β*-LAP, 35 kDa) and liver-enriched transcriptional-inhibitory protein (C/EBP*β*-LIP, 20 kDa), which have common C-termini containing the leucine-zipper and DNA-binding domains but different N-termini resulting in changes to the transactivation domain. C/EBP proteins are regulated by cytokines and play important roles in many cellular processes. Direct physical and functional association can occur between members of NF-*κ*B and C/EBP proteins involving the interaction of the Rel domain of NF-*κ*B with the bZIP domain of C/EBP [33]. Through such interactions, C/EBP can cooperate with NF-*κ*B to regulate cellular promoters, for example, IL-6 and IL-8, and viral promoters, for example, HIV-1 [108], BKV [109], and JCV [31]. However, unlike BKV, the interaction NF-*κ*B/C/EBP*β* regulates JCV transcription in a negative manner. The data reported in the study of Romagnoli and colleagues [31] suggest that interplay between the NF-*κ*B and C/EBP*β* transcription factors may regulate the life cycle of JCV. Since both p65 and C/EBP*β* are regulated by signal transduction pathways activated by cytokines and immunomodulators, cross-communication between these two transcription factors may be important in controlling the balance of JCV latency and reactivation that occurs in response to immunosuppression. Moreover, the regulation of C/EBP*β* occurs at a number of levels, including gene transcription, translation initiation site selection, protein-protein interactions and phosphorylation-dependent changes in DNA-binding activity, potential protein activation and its subcellular localization [33, 34]. Therefore, the C/EBP*β* regulation pathways could also be implicated in the modulation of JCV transcription. Note, all three isoforms of C/EBP*β* (full-length, LAP, and LIP) are expressed in human astrocytic and oligodendroglial cells, which are permissive for JCV replication. From these considerations, it was postulated that cytokines modulating NF-*κ*B and C/EBP*β* activities control JCV reactivation in the brain [35] and that latent virus in oligodendrocytes and astrocytes can be activated by proinflammatory cytokines allowing the expression of viral proteins and viral replication (Figure 2). In highly immunosuppressed individuals, the virus may then be able to infect neighboring cells leading to the spread of virus, because of the lack of an adequate antiviral immune response, and leading to the development of a PML lesion. In particular, the inhibition of CTL migration operated by natalizumab could enhance the spread of virus. On the contrary, few cases are associated with the TNF-*α* blocking although anti-TNF-*α* medications would trigger demyelination. It has been hypothesized that exposure to anti-TNF-*α* might, between other effects, increase survival of autoreactive peripheral T cells penetrating the CNS, produce proinflammatory cytokines such as IFN-*γ*, and cause demyelination [36]. In this scenario, a TNF-*α* blocking could unbalance the fine interaction between NF-*κ*B and C/EBP*β* activities, encouraging a JCV latent state. However, since patients treated with anti-TNF-*α* drugs develop different forms of CNS and peripheral nervous system demyelination, adalimumab and infliximab are recognized treatments for RA, psoriatic arthritis, ankylosing spondylitis, and CD, but not for MS, a demyelinating autoimmune disease treated with natalizumab (Figure 2).

The nuclear factor of activated T cells (NFAT) is another transcription factor family under proinflammatory cytokine control. In particular, NFAT is the primary target of Ca^2+^-calmodulin-dependent serine phosphatase calcineurin, a crucial component of the calcium-signaling pathway that can stimulate the production of inflammatory cytokines in response to extracellular stimuli [37]. Treatment of glial cells with an inhibitor of the NFAT family also inhibited JCV infection, implicating NFAT involvement in the life cycle of JCV. In particular, the activation of NFAT4 in HIV-infected patients due to increasing levels of TNF-*α* and IL-2 may be conducive for JCV infection of the CNS [110]. Moreover, a role for NFAT4 in JCV tropism is possible, as this transcription factor is specifically found in a number of cell types implicated in productive viral infection and latency, including glial cells and bone marrow cells (Figure 2) [29, 111].

Therefore, we can conclude that the JCV life cycle is under the strict control of the immune surveillance, as the transcription of early viral genes is finely regulated by cellular transcription factors under proinflammatory cytokine control.

## 6. Interactions between JCV and Cells of the Immune System: Towards the PMl following a Viral Reactivation from a Latent State rather than a Primary Infection

B cells may play an important role in the pathogenesis of PML, in addition to be a potential site of viral latency. Since it has been suggested that the viral genome rearranges during DNA replication, an attractive model is that these events occur in B cells, since these cells possess the Rag1 and Rag2 enzymes for immunoglobulin gene rearrangements. This hypothesis is sustained by the observation that diverse viral NCCRs, including archetype-like and prototype-like NCCRs, have been found in the blood and bone marrow [112, 113]. Recombination that results in prototype-like viral NCCRs is associated with increased viral activity in glial cells [89]. JCV infection has also been shown to upregulate the DNA damage response [114]. Thus, viral recombination may be explained by chromosomal damage induced by JCV in cells in which recombination and DNA repair mechanisms are active. NCCR recombinations may lead to acquisition of transcription factor binding sites that are important for pathogenesis. A recent example was described in patients receiving infliximab, where an archetype-like NCCR contained sequences that led to TATA box-associated Spi-B sites known to be important for viral replication, while JCV in the urine contained an archetype NCCR sequence [38]. Additionally, as B cells mature, different transcription factors, play a role in increased viral proliferation, are up-regulated. In particular, two factors shown to be important in JCV transcription and regulation: in fact, NF-1X and Spi-B have been shown to be up-regulated in glial cells, B cells, and hematopoietic progenitor cells in which JCV can replicate [11, 57]. Evidence that changes in transcription factors can affect viral transcription could be found in the observation that natalizumab treatment upregulates factors involved in B cell differentiation, including Spi-B [39].

In particular, mutational analysis of the Spi-B-binding sites present in the promoter/enhancer of Mad-4, Mad-1, and archetype clearly demonstrates that these mutated Spi-B sites are important for early viral gene expression in human fetal brain [69]. Spi-B binding sites in the promoter/enhancer of JCV variants are located directly adjacent to TATA boxes, that are essential for the transcription of early and late viral genes. Spi-B is a transcription factor that can cooperate with pRB and TATA-binding protein (TBP) to alter expression of proteins involved in B cell maturation [40, 41]. TBP binds TATA box elements in promoters, and it is a subunit of the basal transcription complex TFIID, which increases RNA polymerase II activity (Figure 2). Recruitment of the TFIID complex to JC viral promoters by Spi-B and TBP is an attractive model for the activation of JCV gene expression. The proximity of TAg-binding sites, TATA boxes, and Spi-B-binding sites on the JCV promoter/enhancer suggests that TAg and Spi-B may cooperate in recruitment of transcriptional apparatus components in immune cells, similar to the cooperation of TAg and Oct-6/tst-1/SCIP in glial cells (Figure 1) [115]. Interestingly, initial events of JCV infection occur in the absence of TAg protein, suggesting that other factors regulate this process.

Moreover, archetype JCV is replication incompetent in tissue culture and is associated with asymptomatic persistence in the kidney of normal healthy individuals [116]. Although archetype-like JCV sequences have been isolated from PML tissue, these viral sequences consistently contain mutations and deletions [117]. The sequence encoding the inactive archetype Spi-B site, AAAAGGGAAGG**T**A, is conserved among viruses isolated from the urine of healthy individuals [89, 118–120]. However, the novel observation of one such point mutation that creates an active Spi-B-binding site (AAAAGGGAAGG**G**A), directly adjacent to the archetype TATA box, which convert archetype to a biologically active virus, capable of expressing both TAg mRNA and TAg protein significantly increased levels over wild type [121]. Importantly, similar mutations that create active Spi-B-binding sites adjacent to the TATA boxes of archetype-like viruses have been described in the brain of a patient with cerebellar atrophy [122] and in intestinal biopsies of CD patients treated with infliximab [38], suggesting that this type of mutation is supported during dissemination in the host (Figure 1). However, future analyses of alterations and acquisitions of unique transcription factor-binding sites naturally occurring in patients will probably offer more insight into the role of these factors in viral pathogenesis. 

Moreover Marshall and colleagues [121] have identified Spi-B-binding sites on JCV NCCR that bound Spi-B protein expressed in cells derived from human fetal brain, but not from the immune system. These results indicate a difference between Spi-B isoforms expressed in the brain and B-cells. This work has demonstrated that Spi-B binding to the viral promoter contributes to early viral gene expression in astrocytes; however, Spi-B is naturally expressed at high levels in developing B cells. B-cells have been shown to support the low levels of JCV infection and are likely carriers of infectious virus to the brain during reactivation and dissemination leading to the development of PML. The activation of Spi-B gene expression in transitional B cells that contain latent virus may be an important step in viral reactivation. Further investigation into the molecular interactions that occur between Spi-B, protein cofactors, and the JCV NCCR in cells that support latent (haematopoietic progenitors and B-cells) and productive (astrocytes and oligodendrocytes) infection will offer additional insight into molecular pathogenesis, reactivation from latency in lymphocytes and the development of PML.

## 7. Conclusion

Finally, several pathways finely regulated determine the JCV reactivation from its latent state and the JCV NCCR recombination, leading to the emergence of neurovirulent variants. However, the host immune system plays a decisive role in facilitating or less the expression of particular cellular transcription factors, which are essential to the virus for its reactivation and its subsequent productive infection into permissive cells. The same pathways that lead to the expression of specific transcription factors determine the cell permissiveness to JCV infection, and, as it is known, among these pathways, the most important are under the control of the host immune responce (NF-*κ*B, C/EBP*β*, and Spi-B).

Morover, it is important to note that B cells may carry JCV across the BBB: in fact, PML was first associated with B cell lymphoproliferative disorders [47], and natalizumab treatment-associated PML occurs concurrently with the mobilization of lymphocytes from the bone marrow to the periphery [8]. Additionally, HIV-induced depletion of lymphocytes in the periphery may lead to the mobilization of lymphocytes from the bone marrow to the periphery, and PML may be “unmasked” after the reconstitution of the immune system by HAART treatment [123]. In addition, JCV may cross the BBB as free virus to initiate infection of oligodendrocytes. JCV may also infect microvascular endothelial cells and thereby cross into the brain [124]. In this scenario, the events that must occur to cause JCV lytic infection of the oligodendrocytes in the brain are (i) the host immune system impairment, (ii) the viral NCCR rearrangement to increase viral transcription and replication in both B cells and glial cells, (iii) the upregulation of DNA binding factors that bind to recombined NCCR sequences in infected hematopoietic progenitor, B cells, and/or glial cells, and (iv) the migration of free JCV or carried by B cells across the BBB. In particular in the brain, both NFAT4 and NF-*κ*B expressions can also increase JC early transcription in response to TNF-*α* [29, 30]. Additionally, C/EBP*β* has been proposed to repress early transcription and is under proinflammatory cytokine control [31]. Therefore, oligodendrocytes and astrocytes, which harbor JCV in a latent state, could be strictly under the control of powerful cytokine-signaling pathways, in particular those of proinflammatory cytokines such as TNF-*α*. For this reason, in individuals treated with immunomodulatory drugs, an impairment of the immune surveillance of the CNS could be an important risk factor for the JCV reactivation and the PML onset. Therefore, it is important to focus all our efforts to find the cellular pathways, finely regulated by the host immune system, that lead to the reactivation of the virus in conditions of severe immunosuppression, as it is virtually impossible to control the JCV infection.
